# The psychological process and emotional cognition of children's tourism experiences in Chinese family culture

**DOI:** 10.3389/fpubh.2022.960534

**Published:** 2022-08-11

**Authors:** Xuanxuan Guo, Tao Liu

**Affiliations:** ^1^Management College, Guangdong Polytechnic Normal University, Guangzhou, China; ^2^Department of Sociology, Hangzhou Dianzi University, Hangzhou, China

**Keywords:** psychological process, emotional cognition, children tourism experience, children's perspective, drawing projection

## Abstract

The family cultural environment affects children's cognitive development and socialization processes, and different family cultural environments lead to differences in children's tourism experiences. The current research on children's tourism experiences demands a shift from the families' perspective to that of children's perspective. In response to this, grounded on the cognitive development theory, this paper, from the perspective of children's memorable parent-child tourism experience, uses 321 children's drawings to project the tourism elements, people, activities, scenes, and colors that children perceive from travel, reveals the main cognitive contents of children's parent-child tourism experiences. Furthermore, this paper analyzes the influence of family cultural background on children's tourism experiences through interviews with children. Our results show that with the growth of age, children's perception of elements changes from the macro level to the micro level, and the contents they perceive change from concrete to abstract. In addition, children have an acute perception of people and are impressed by novel activities during travel. They adapt well to changes in travel scenes and prefer bright and vibrant colors during trips. Therefore, we recommend the design of appropriate tourism products that combine the characteristics of children's experiences when offering parent-child travel programs, as well as upgrading the market of parent-child tourism experiences through novel activities.

## Introduction

Parent-child tourism is a family tourism activity centered on children's experience. It aims to enhance the parent-child relationship, increase children's generic skills (1), promote children's socialization, and exert parental influence. As a subset of family tourism, parent-child tourism remains an issue for further research compared to mature family tourism research, such as parent-child tourism experience. Children are not miniature versions of adults, and they cannot be treated as adults in the parent-child tourism experience. Instead, they have their own emotions and perceptions. In the past decade, some scholars have explored that children have unique perspectives and are capable of presenting them (2–4). Similarly, this study emphasizes the unique role of children in parent-child tourism scenarios. It advances children's emotional experiences research through exploration of Chinese children's perceptions of memorable parent-child tourism.

A comprehensive review of children's emotional experiences research suggests that most studies have been conducted in daily or familiar scenarios (5–7). However, there are considerable differences between daily and tourism scenarios (8, 9). For example, Zhong and Peng (10) suggested that children have a different emotional experience in tourist world. They consider recreational tourism as an upgraded version of daily play that is more interesting. Moreover, most studies on children's tourism experiences have been conducted in western contexts. However, there are some kind of differences between western and Chinese cultural (11, 12). For instance, Chin et al. (13) points out that the central governments to the COVID-19 event have revealed salient cultural difference, people in China with more group-centred and collectivist cultures. Leisure and life enjoyment, filial piety and relationship, self-fulfillment, righteousness, and humanity are Chinese cultural value factors (14). In addition to the potential differences, the increasingly important status of the children in the Chinese family culture should not overlooked (15, 16). As a result, it is necessary to study children's emotional experience in tourism scenarios, especially in the context of Chinese families culture.

Understanding child's tourist world seems not easy. A child's world has its special values and meanings, and the attempt to analyze a child's world from an adult perspective is actually a compulsion to replace the objective state as it is with the subjective state as it ought to be (17). Educational behaviors guided from an adult perspective have widened the gap between the adult world and the child's world. In life, what adults give to children may only be the wishful thinking of adults, and this can easily cause physical and psychological harm to children (56). It has become increasingly important to make understand children. In view of mentally immature children cannot convey a complete inner schema to adults, which makes it challenging to understand them. Therefore, the childlike aspects of children's minds require appropriate means to investigate their experiences. To solve this problem, this study attempts to use children's drawings to explore what kinds of tourism experiences are memorable for children and to explain children's perceptions and experiences of parent-child tourism. The study also identifies the specific content and emotional cognition of the experience, thereby proposing suggestions for the effective enhancement of the parent-child tourism experience.

In brief, the purpose of this study is to explore what Chinese children's views of parent-child tourism experiences. The study's four objectives are as follows: (a) investigate the parent-child tourism experience of children in the context of Chinese family culture; (b) explore the cognitive content of children's memorable parent-child tourism experiences; (c) identify the regular characteristics of children's parent-child tourism experiences; (d) put forward countermeasures and suggestions to improve children's tourism experiences.

The main contribution of this study is its description children's emotional cognition in the context of parent-child tourism, which expands from the daily scenarios to the tourism scenarios, and its exploration children's experiences in parent-child tourism through drawing projection, which makes up for the lack of children's perspective in tourism experience research. In addition, this study have been conducted in Chinese cultural context, including both urban and rural children, in order to better understand the diversity of children's tourism experiences. The research conclusion is helpful in understanding children's tourism experiences within the Chinese cultural background. At the theoretical level, the study enriches the theory of children's tourism psychology and helps deepen the understanding of non-western children's tourism experiences. At the practical level, this study can provide consumer insights for children's tourism service providers and for designers and marketers of parent-child tourism products.

The structure of this study is as follows. In the literature review section, we summarize the children and parent-child tourism and the technique of children's drawing in tourism research. In research design, we describe our research methodology, study area, and data collection. The research findings section presents the results of our data analysis. Finally, in conclusions and implications, we discuss the key findings of the research and put forward theoretical and practical implications.

## Literature review

In order to provide a context for the children's tourism experiences research, parent-child tourism and the role of children in this phenomenon will be discussed, followed by a consideration of the technique of children's projective drawing in tourism research.

### Children and parent-child tourism

Parent-child tourism is a scenario in which children and their parents take trips together. As opposed to trips in which children take part independently (organized by schools or institutions), parent-child tourism places a dual emphasis on children in the context of family-oriented self and self-oriented self. The parent-child tourism experience comprises the psychological process and feelings of children arising from the interaction between themselves, the tourist scene, and the family scene. Such experiences are characterized by the body of the child as the axis, emphasizing the organic unity of brain, body, and environment. It can be supposed that the core subject of the parent-child tourism experience is the “child,” the parental experience is generally secondary.

Most studies on tourism experience are based on the hypothesis that “individuals have the freedom to choose their preferred destinations and make rational decisions,” but this classic hypothesis is usually not applicable to the tourism experience of families with children (18). The family-based nature of parent-child tourism makes it difficult to consider only the individual without considering the family unit in which the individual is involved (19). Parent-child tourism is a scenario in which children and their parents take trips together. As opposed to trips in which children take part independently (organized by schools or institutions), parent-child tourism emphasizes children's tourism experiences under the family background. When vacationing with children, adults sacrifice their own needs and preferences for the joy of their children (20). That is, in the context of parent-child tourism, parents are less interested in seeking novelty and more interested in spending quality time with their children, making up for the lack of emotional moments in their daily lives, creating shared memories, and fostering their children's growth and development. This family consumer mentality, which reflects the family cultural, affects the children's tourism experiences (15, 21). The term “family culture” refers to subjective factors such as the knowledge, thoughts, values, attitudes, and behavior styles of family members (22). Different family cultural environments lead to differences in children's tourism experiences. Khoo-Lattimore and Yang (12) pointed out that Eastern countries with strong family values under the influence of Confucian culture can provide better tourism experiences for children. Qiao et al. (23) found that the left behind children's tourism experience is lower than that of non-left behind children in the situation of traveling with parents. Yang and Lau (24) found that families with strong educational motivation have better learning experiences in cultural heritage tourism destinations.

Despite the extreme importance of children in parent-child tourism, their voices seem to be ignore and under-valued by researchers (19). Most studies that have investigated issues related to the parent-child tourism experience have done so from the perspectives of parents. Li et al. (25) analyzed parents' blogs and suggested that the tourism experiences of children from birth to 4 years of age are characterized by two main features: the pro-nature properties of enjoying the outdoors and unconscious participation in social development. Hassell et al. (26) used a parental perspective to interpret children's experiences and experiential values while camping in national parks. Lehto et al. (27) examined children's tourism experiences gained in overseas summer camps from the perspective of parental expectations. Gram et al. (3) explored the complex emotions that different characters perceive in family tourism from multiple family-child perspectives. Scholars such as Zhong et al. (28), Li et al. (29). and Xie et al. (21) have initiated their research on tourism from the perspective of children. These studies have brought more attention to the status of children in tourism, leading researchers to focus on children's experiences in different tourism situations. Although scholars have affirmed the role and influence of children in parent-child tourism, their studies still neglect children's subjective initiative. The psychological processes and emotional cognition of children's self-based experiences in parent-child trips should be further investigated in the context of “family-oriented self.”

A review of children and parent-child tourism research suggests at least two research gaps. Firstly, insufficient attention to children's voices and psychology in tourism scenario by researchers. It is evident that children's voices cannot be replaced by their parents' or other stakeholders' perspectives (30, 31). It needs to be acknowledged that parents and children have different views, although in the same scenario (10, 32). So it is necessarily to use appropriate methods and scenario to understand children's tourism views. Secondly, from the perspective of cultural geography, most studies on children tourism has been set in western cultural background (12, 33, 34). Currently, as Chinese authorities have introduced a set of guidelines, such as to ease the burden of excessive homework and off-campus tutoring for students undergoing compulsory education, and to open the third child, China parent-child tourism market is booming (35). As a result, research on children's tourism experiences should be conducted in the background of Chinese family culture.

### Children's drawing in tourism research

As a means of studying children's tourism experiences, drawing can reflect children's cognition of the world and their psychological developmental characteristics (36). Drawing is also a concrete expression of children's emotional expression (37). Children's real perceptions of the natural world are processed by their own perceptual information and conveyed, expressed, and translated onto the paper through the language of drawing (38). This is a cognitive process in which the external scene is viewed and expressed as one.

Since the 1940's, psychological researchers have agreed that drawing can reflect a person's emotions, states of consciousness, and personality traits, resulting in the subsequent introduction of the term “projective drawing.” Psychologists and psychoanalysts have begun to conduct research using projective drawing tests. Drawing can be helpful in the study of special groups in that it can project the mental worlds and map the subjective experiences of those with potential expression disorders (39). Drawing is a special “language” for children. Through colors, lines, figures, shapes, layouts, and other forms of expression, a picture with internal logic is formed to reveal children's emotional characteristics, psychological emotions, and cognitive levels. When children describe the world they perceive on canvas through the language of drawing, it is mainly based on their realistic portrayal of objects in nature and the processing of their perceptions.

Children's drawing is relatively rare in tourism research (30), which has been widely used in the research of children in education, psychology, and art fields (40, 41). However, this approach is one of the best ways to overcome the bottleneck of traditional methods for children's tourism research. Hay (33) pointed out its a challenge to get authentic information from young children as research subjects, which is the reasons for the missing of children's voices in family tourism research. Children's drawings offer the most direct route to children's hearts and can compensate for the shortcomings of research methods, such as interviews and diaries. A number of scholars have used this method to examine children's attitudes toward tourism (42), their travel behavior (30), their family tourism experiences (15, 31), and their perceptions of the socio-spatial environment of tourism (43). In conclusion, it has become a consensus among scholars to interpret the world of children's tourism through children's drawings.

Synthetically, the deficiency of research on children's tourism experiences with their parents, particularly in the context of Chinese families culture, calls for more academic efforts in this area. Given the rising importance of parent-child market in China, our study was conducted in the parent-child scenario, different from the daily scenario. Its uses children's projective drawing and examines the cognitive representations of Chinese children's contents of parent-child tourism experiences.

## Research design

### Research methodology and objects

The technique of children's projective drawing was adopted for this study, supplemented by brief interviews. In accordance with the theory of the drawing elicitation interviews method, the children first drew a picture of their “favorite/most memorable trip with father/mother.” After the children completed their drawings, the researcher, with the assistance of teachers, asked the children to take between 3 and 8 min to briefly explain what they had drawn. The children answered the following questions: (1) What did you draw? (2) Why did you draw this? (3) Give a brief description of the trip; (4) How did you enjoy the trip? Moreover, the researcher recorded their answers.

We studied elementary school children from the first to sixth grades, mainly between the ages of 6 and 12. We chose children from this age group because preschool children are limited by their cognitive levels and sports ability (44). They play an extremely passive role in tourism activities, so it is hard to use them to accurately perceive the tourism experience. In contrast, children in middle school are mature; but they are prone to external interference, and the tourism experience is not pure. Children in primary school, however, not only have mature cognitive judgment ability but can also maintain relatively pure experience perception (45). In short, unlike toddlers, children in elementary school have begun to think independently and understand social actions. They can communicate with others independently to a certain extent and describe a story or scene almost completely.

### Study area

This study was conducted in Guangzhou, Shenzhen, and Maoming in Guangdong Province, and included both urban and rural areas. Most of the existing studies related to parent-child tourism have been conducted in urban areas, and the data collected are mainly on urban children (15), neglecting children in rural areas to some extent. Existing research calls for removing the “binary divide” between rural and urban areas, arguing that the goals of basic education in rural and urban areas should be the same (46). As a result, we need to pay attention to rural children, who also participate with their parents in parent-child tourism and receive the corresponding experiences. According to the relevant parent-child tourism statistical survey, residents of Guangdong are most concerned with travel and rank near the top in China in terms of their commitment to traveling with their children. At the same time, parent-child tourist attractions, such as Chimelong Tourist Resort, are also popular among tourists from both inside and outside Guangdong Province. In addition, the various regions of Guangdong Province are unevenly developed, with the eastern, western, and northern parts being much less economically developed than the Pearl River Delta region. In this study, Guangzhou, Shenzhen, and Maoming were selected as the main locations for the survey on parent-child tourism experiences. Data were collected from children of different backgrounds, including those from municipal key public schools, municipal non-key public schools, and county-level public schools. Therefore, the study comprehensively reflects the parent-child tourism experience of urban and rural children and provides a relatively comprehensive picture of the research question.

Three elementary schools in Guangdong Province were selected as the primary survey sites. On the one hand, children's data can be collected in schools in a way that reduces heuristic interference of parents on children's thinking. On the other hand, the parent-child tourism experiences of children from different family backgrounds can be more comprehensively captured from different levels of elementary schools. In China, educational resources is geographically unbalanced, schools in nicer areas serve children with better family backgrounds (47). For this paper, we selected elementary schools as research sites according to geographic location, school level, and possibilities for maximizing the availability of data at all levels.

The basic information for the three elementary schools is shown in Table 1. Situated in Baoan District, Shenzhen City, Guangdong Province, School 1 is a long-established municipal key public school with a favorable location and superior educational resources. The school district system in China has contributed to an imbalance of educational resources to some extent. Good school districts are often populated by better-off families, so most of the students at this school come from affluent families. School 2, located in Tianhe District, Guangzhou City, Guangdong Province, is a municipal non-key public school with a relatively short history of operation and moderate size. The students at this school are mostly from middle-class families, and their parents are mainly new immigrants to the city. School 3 is situated in Diancheng Town, Dianbai District, Maoming City. It is a small, county-level public school in a remote location. It has a rural student population and parents with lower average salaries and education levels than those of parents in schools 1 and 2.

**Table 1:** Basic information of the three schools.

**Category**	**School 1**	**School 2**	**School 3**
Location	Baoan District, Shenzhen	Tianhe District, Guangzhou	Diancheng Town, Maoming City
Nature	Municipal key public elementary school	Municipal non-key public elementary school	County-level public elementary school
School history	1947	2002	1978
Dominant type of students	Urban household registration	Urban household registration	Rural household registration

### Data collection

Most parents choose to take trips with their children during the summer vacation and the National Day holiday. Consequently, we collected our data within 2 months of the end of summer vacation so that children would still vividly remember the trips taken. At the same time, given moral considerations, the content and requirements of the study were made known to the children's parents in advance through the social media groups of the school classes. If parents did not support their child's participation in the study, the child was not surveyed.

Over the course of 2 months, we collected 390 children's drawings (130 from each school) for the study. Based on the content of the drawings and interviews, samples that did not fall into the category of parent-child tourism, such as trips to the neighborhood swimming pool, trips without parents, and field trips, were excluded. Moreover, samples in which children did not describe their drawings well were excluded. The final number of valid samples was 321, and the data validity rate was 82.30%. To facilitate statistical analysis, the 321 drawings were simply coded. The coding process was based on grade, serial number, and gender. For example, the code “1-01X” would represent the sample of a child in the first grade of elementary school, with the serial number 01, of the female gender. The descriptive statistics of the participating children are shown in Table 2. The information about children's experiences and types and times of trips with their parents was sent to the parents by the teachers of each class for statistical and reconfirmation reasons.

**Table 2:** Descriptive statistical information of valid samples in the survey (*N* = 321).

**Attribute**	**Category**	**Number**	**Frequency**
Gender	Male	150	46.73%
	Female	171	53.27%
School	School 1	120	37.38%
	School 2	116	36.14%
	School 3	85	26.48%
Grade	Grade 1–2	137	42.68%
	Grade 3–4	77	23.99%
	Grade 5–6	107	33.33%
Trip type	Mom, dad, and child	169	52.65%
	Mom and child	98	30.53%
	Dad and child	18	5.61%
	Three-generation trip	36	11.21%
Time for traveling	Summer and winter vacations	186	57.94%
	Minor vacation	66	20.56%
	Weekend	58	18.07%
	Other	11	3.43%
Tourism experience	Experience traveling abroad (excluding Hong Kong, Macao and Taiwan)	30	9.35%
	Experience outside Guangdong Province (including Hong Kong, Macao and Taiwan)	171	53.27%

As shown in Table 2, the number of girls and boys participating in the survey was almost equal. There were 120 valid samples in School 1, with a validity rate of 93.30%, and 85 valid samples in School 3, with a validity rate of 65.38%, which is related to the gap in resources between urban and rural areas. Children in urban areas are exposed to more recreational activities, while children in rural areas have limited spiritual and material resources, and their parents are generally working far away from home. Therefore, children in rural areas have misconceptions about parent-child tourism and describe it in a way that easily confuses regular daily activities with traveling. Moreover, children in rural areas are weaker in language expression than children in urban areas, resulting in lower sample validity. The most memorable parent-child trips, as described by children, were those on which they traveled with their mothers and fathers, followed by trips on which they were accompanied by their mothers. Inter-generational family trips (11.21%) and those on which they traveled with their fathers (5.61%) were less frequently depicted in the drawings.

## Research findings

In accordance with the rules of drawing elicitation interviews, this paper focuses on children's descriptions of past trips with family members in the analysis of their drawings, rather than on the artistic merit of the drawings. The level of drawing did not affect the study results. By identifying what the 321 children drew, we analyzed the relationship between leadership and five other factors: perception of tourism elements, perception of people in travel, experience of tourist activities, experience of tourist scenes, and experience of travel colors.

### Perceptions of tourism elements

The tourism elements in the drawings are the most intuitive perceptions of children and reflect their core memories of trips with their parents. For the statistical analysis, the children were first divided into three groups: lower, middle, and upper grades. The lower grades category included first and second graders, the middle grades included third and fourth graders, and the upper grades category was comprised of fifth and sixth graders. The analysis revealed that the memorable pictures of children's trips with their parents were mainly composed of people, natural scenery, animals, architecture, food, plants, boats, words, and vehicles.

The cumulative statistics of the above tourism elements in the drawings are shown in Table 3. People (82.55%) and natural scenery (74.77%) were most frequently represented in children's drawings, followed by words (43.30%), plants (39.56%), architecture (37.07%), and animals (36.76%), and less frequently by food (16.82%), boats (15.58%), and vehicles (15.64%). In terms of grade level, children of different ages are concerned with different tourism elements. First, the attention to people on trips decreased with grade level, while attention to natural scenery increased with grade level. This shows that children's descriptions of parent-child tourism experiences gradually shifted focus from people on trips to natural scenery as they grew older.

**Table 3:** Statistical analysis of tourism elements in children's drawings.

	**People**	**Natural scenery**	**Animals**	**Architecture**	**Food**	**Plants**	**Boats**	**Words**	**Vehicles**
Lower grades	119	86	45	58	11	56	21	38	19
	86.86%	62.77%	32.85%	42.34%	8.03%	40.88%	15.33%	27.74%	13.87%
Middle grades	66	57	30	33	17	25	11	34	10
	85.71%	74.03%	38.96%	42.86%	22.08%	32.47%	14.29%	44.16%	12.99%
Upper grades	23	23	23	23	23	23	23	23	23
	74.77%	90.65%	35.51%	26.17%	24.30%	42.99%	16.82%	62.62%	21.50%
Total	265	240	118	119	54	127	50	139	47
	82.55%	74.77%	36.76%	37.07%	16.82%	39.56%	15.58%	43.30%	14.64%

Second, the frequency of animals, plants, and boats remained similar in the lower, middle, and upper grades, and did not fluctuate with grade level. However, children in the upper grades showed significantly more concern with food and vehicles, which were more evident in the drawings. This indicates that children's perceptions changed from macroscopic to microscopic, from common animals and plants in initial trips to more specific and detailed food and vehicles in parent-child tourism, and children gradually became interested in modes of travel and food experiences. Finally, 62.62% of children in the upper grades tended to describe the pictures of their trips with their parents with words, much higher than the 27.74% in the lower grades, indicating that children's perceptions of tourism elements moved from concrete to abstract.

### Perceptions of people on trips

People on trips are the various people with whom children come in contact during parent-child tourism. These are mainly fellow travelers, or the people who travel with children, such as their parents, elders, classmates, or tour mates. During the parent-child tourism experience, children inevitably interact with others, which enhances their interpersonal communication and leadership skills. In our analysis of tourism elements, we found that more than 80% of children included people in their unforgettable memories of parent-child tourism, so we analyzed the number of people in the drawings for statistical description, as shown in Table 4.

**Table 4:** Statistical analysis of persons in parent-child tourism.

	**0 persons**	**1-2 persons**	**3-4 persons**	**More than 4 persons**	**Number of respondents**
Lower grades	17	23	72	25	137
	12.41%	16.79%	52.55%	18.25%	
Middle grades	10	18	29	20	77
	12.99%	23.38%	37.66%	25.97%	
Upper grades	29	21	34	23	107
	27.10%	19.63%	31.78%	21.50%	
Total	56	62	135	68	321
	17.45%	19.31%	42.06%	21.18%	

**Figure 1:**
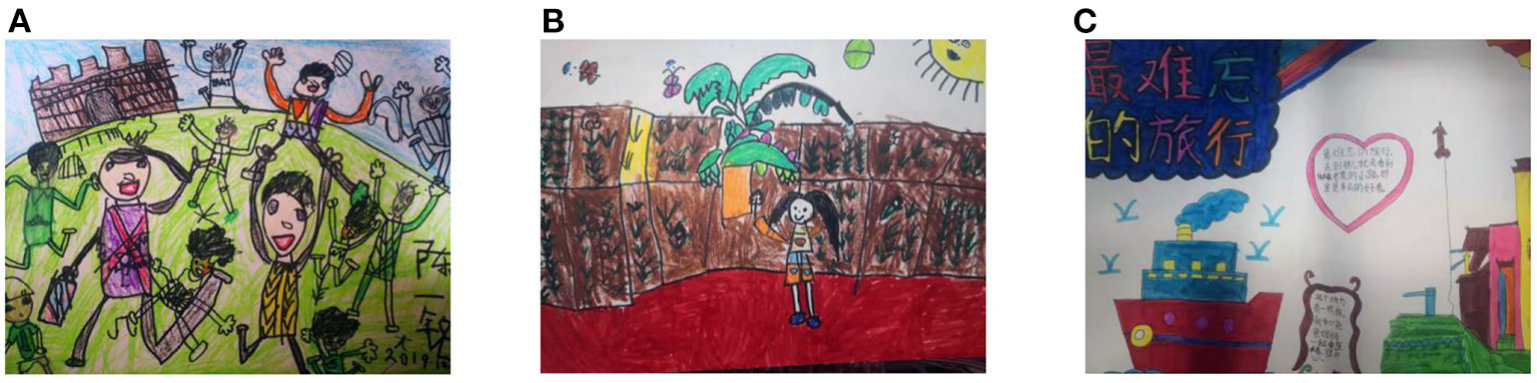
People perceive in children's drawings **(A)** Group (1-54Y); **(B)** Individual (3-15X); **(C)** Objects in the tour (5-25Y).

The younger the child, the more he or she preferred a more crowded travel style (≥3 persons), whether it was mother, father, siblings, or even strangers during the trip, which left a deep impression on the younger children and was reflected in their drawings. As seen in Figure 1A, the child numbered 1-54Y explained his drawing in the following way:

“*I went to Chenzhou with my parents, and I met many new friends, especially the younger boy from my father's colleague's family. I played with toys, ate yummy snacks, and took pictures with him. How lively it was, and it was not lonely because there were so many people.” (A,1-54Y)*

This quote reveals that children in the lower grades like to go on trips with many people and that they value contact with others during a trip. Their core cognition is simple and concrete, and their self-cognition is further influenced by the behavior of the other. As children progress in grade, age, and experience, they begin to develop logical computational skills and rational thinking. Therefore, in addition to having deep memories of crowded activities, children in the middle and upper grades tend to be more interested in self-activity or in the objects themselves, such as tourist symbols, buildings, or landscapes during a trip. After depicting an individual activity scene in which she pulled up radishes in a farm field, the middle-grade child in Figure 1B said the following:

“*This is a farming world, and I found a papaya tree with lots of fruit on it.” (B, 3-15X)*

In Figure 1C, a child in the upper grades attached more importance to the description of objects on a trip.

“*What I remember most about the trip is how nice it is to walk there and see the paths like those of my hometown. There is a boat in this beautiful town, and I had a lot of fun riding on it with my mom and dad.” (C, 5-25Y)*

From this, we can see that children in the middle and upper grades begin to develop the ability to think independently during family trips.

**Figure 2:**
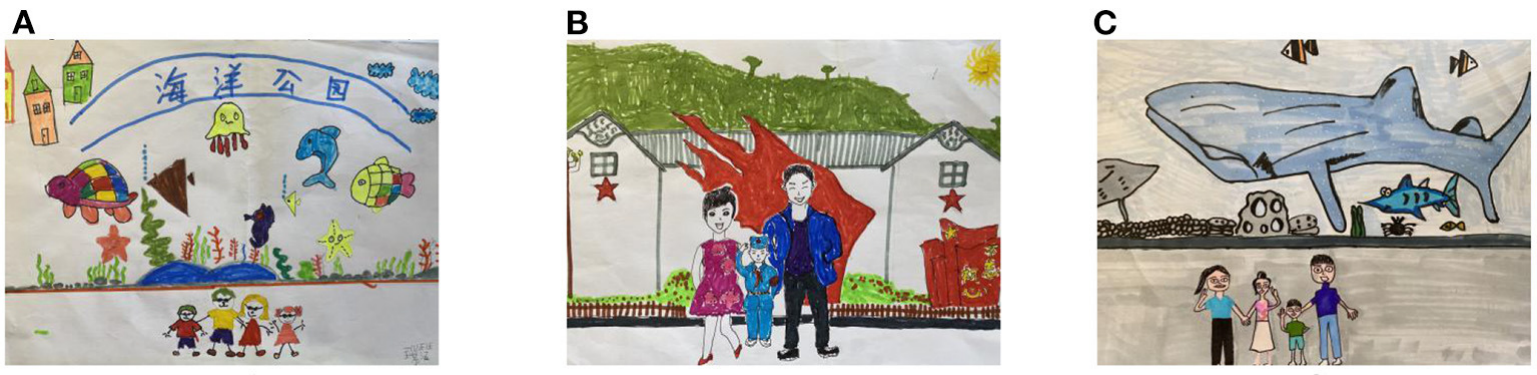
Parental companionship in children's drawings **(A)** Lower grader (1-08Y); **(B)** Middle grader (3-15Y); **(C)** Upper grader (5-05X).

Overall, children are sensitive to the people with whom they come in contact on trips with their parents. More than 40% of the children presented three or four people in their drawings, which is closely related to the composition of modern nuclear families and reflects the children's emotional attachment to their parents and siblings. Figure 2 shows the children's tourism memories with their families in front of major tourist attractions. One child said the following:

“*I went to the ocean park with my mom, dad, and sister. There were a lot of marine animals there, and my sister and I were very happy. I like it there, and I learned about many kinds of fish” (A, 1-08Y)*.

“*Mom and Dad took me to the Haifeng Xinshan Revolutionary Memorial Museum. They told me to learn from the revolutionary martyrs. My mom dressed me in a military uniform, and I saluted” (B, 3-15Y)*.

“*Mom and Dad took my brother and me to the aquarium. It was a rare trip for us to go out together, and my mom told me to take care of my younger brother. We also took a picture with a shark as a souvenir” (C, 5-05X)*.

It is evident that family members are important companions for children in parent-child tourism and can influence children's cognitive, emotional, and intellectual memories. Children of all ages include their parents and siblings in their world, and they are an integral part of memories in parent-child tourism. In 21.18% of the drawings, children showed people on trips other than their parents and family members, and they described the actions of other travelers involved in similar experiences, such as playing games at amusement facilities, sunbathing on a beach, or watching the scenery from a ship. In addition, 17.45% of the children had no characters in their drawings, and they focused more on the objects of the trip. In conclusion, the people in the children's drawings consisted mainly of the core members of the family, and the interaction between these members had an effect on the formation of children's unforgettable parent-child tourism experiences, which in turn influence children's tourism experience benefits.

### Experiences of tourist activities

Although adults pay attention to the emotional value and educational value of parent-child tourism, children pay more attention to play in travel, and play needs to be realized through interesting tourist activities. Tourist activities are major channels of dialogue and interaction between children and the outside world. Through tourist activities, children engage in dialogue with the natural landscape and social culture and learn about the world of tourism, which enriches their inner beings and broadens their horizons. Our data show that most of the children's drawings show a variety of tourist activities. The analysis of tourist activities presented in children's drawings can reveal the original playful nature of children in tourism.

According to the drawings, unforgettable tourist activities for children can be divided into three categories: recreation and leisure, sightseeing and exploration, and cultural and historical. The recreation and leisure activities include rafting, hot springs, playgrounds, horseback riding, snowman building, and other simple activities. Sightseeing and exploration activities include certain modes of thinking, such as scenery viewing, exploration, architecture appreciation, and plant and animal observation. Historical and cultural activities are those that take place in tourist destinations with historical and cultural attractions, such as ancestor worship, village experiences, and visits to Tiananmen Square.

**Figure 3:**
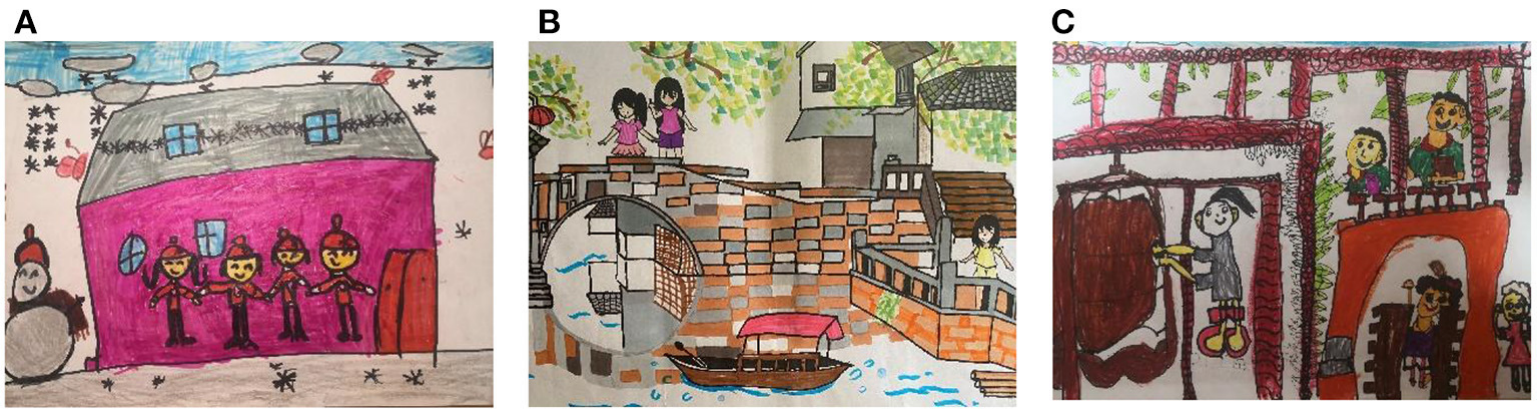
Parental companionship in children's drawings **(A)** Recreation and leisure activities (1-44Y); **(B)** Sightseeing and exploration activities (5-23X); **(C)** Cultural and historical activities (5-05X).

While children in the lower grades find simple recreational and leisure activities most memorable, sightseeing and exploration activities, as well as and historical and cultural pursuits, gradually become more attractive to children in the upper grades. From the children's drawings, we can see that as the grade level increases, children's impressions gradually shift to participatory, experiential, and knowledge-based cultural and recreational activities, such as experiencing high-tech or dancing with local people. This may be related to the evolution of children's cognitive levels, from superficial visualization of natural landscapes to deeper rational cognition. Children are most interested in participating in tourism activities that are different from their everyday lives and that appeal to their curiosity and desire for novelty. Figure 3 illustrates three types of tourism activities. The children described each of the drawings as follows:

“*I went to America with my mom and dad. When I got up in the morning, I found it snowing. It was the first time I had seen snow, and I was so happy to see the snowflakes. I also built a snowman, and I saw many children and adults enjoying themselves in the snow. My mom even taught me how to build a snowman” (A, 1-44Y)*.

“*My mom and I were traveling in Zhouzhuang. We were mainly there for sightseeing. Zhouzhuang is very special, with small bridges and flowing streams. We stayed in an inn, which was completely different from the big hotels, and I remember the owner was very welcoming and amused me” (B, 5-23X)*.

“*I went to the Confucius Temple with my parents. My mom told me that the Confucius Temple is part of traditional Chinese culture, and the Analects I memorized as a child were written by Confucius. What impressed me most was that there was a big bell there, and when I went to ring it, the sound was so loud that it scared me” (C, 5-05X)*.

Although children's feelings vary concerning different tourism activities, children are constantly absorbing new knowledge schemas from the outside world while having fun and playing. In the ongoing interaction with the environment, people, and things, children are also learning informally about nature and what is beyond the textbook, such as seeing snow for the first time, observing small bridges and flowing streams, and experiencing Confucian culture, all of which can lead to unforgettable parent-child tourism experiences.

### Experiences of tourist scenes

Children paint pictures of parent-child tourism with their brushes as a reflection of tourist scenes in their memories. Children's drawings are generally classified into five types: imaginative drawings, fantasy drawings, story drawings, memory drawings, and sketching drawings (14). On this basis, Wang et al. (31) classified children's tourism drawings into the categories of realistic, storytelling, and imaginative. Croce et al. (48) classified tourism stories as factual or imaginative in their study of children's tourism experiences. In summary, this paper argues that the scenic content of tourism drawings is the reproduction of memories in parent-child tourism, and the scenes are classified into realistic scenes, story-based scenes, and associative scenes. Realistic scenes are the mirror of the objective items in the travel process, what is “seen” on the tour. Story-based scenes are drawings that use the plot or clues of a trip as the material for expression, and stories can induce children's true feelings. Associative scenes are imaginary images that are processed by the brain (i.e., images that are further associated with the travel context, such as the presentation of elements from fairy tales).

**Figure 4:**
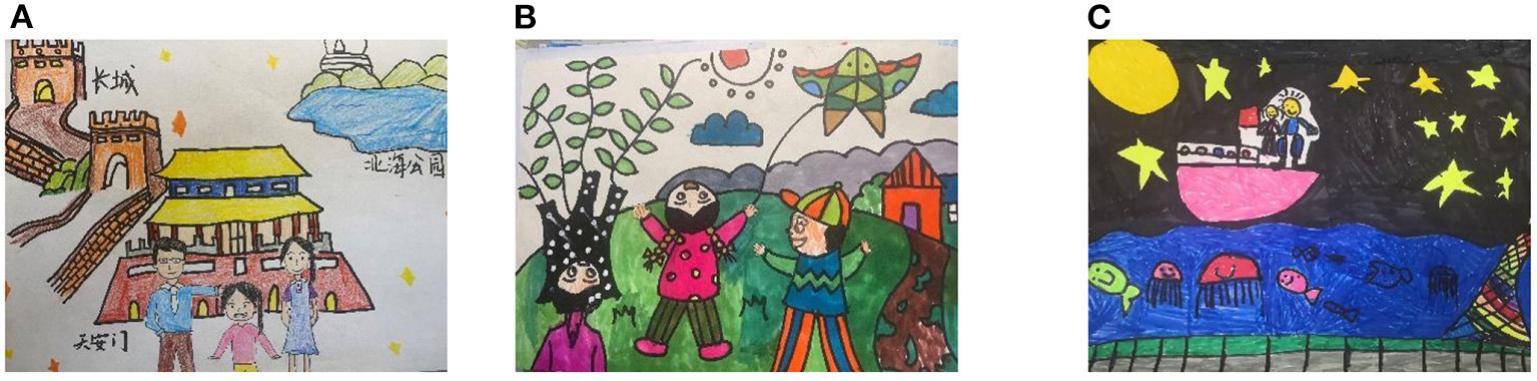
Tourist scenes in children's drawings **(A)** Realistic scenes - A tour in Beijing (5-20X) (1-08Y); **(B)** Story - based scenes - Flying kites in the park (1-10X); **(C)** Associative scenes - Night tour (4-19Y).

From Table 5, we can see that more than half of the children's drawings depict realistic scenes, story-based scenes account for about 30% of the drawings, and associative scenes account for only about 13% of the drawings. In terms of grade level, drawings of realistic scenes decreased from lower to upper graders, from 60.58 to 51.40%. The number of drawings with story-based scenes was about equal in all grade levels, but the highest percentage was in the upper grades (30.53%). The drawings of associative scenes were more in the middle and upper grades, about 15%. Figure 4 shows the children's drawings in each of the three scenes. Children's perceptions and memories of trips with their parents are primarily based on concrete and objective items, such as Tiananmen Square, the Great Wall in Beijing, the Presidential Palace, or the monuments in Nanjing. At the same time, elementary school-aged children have the ability to think about stories and recount and reflect on their trips. Some children described the story of flying a kite in the park or the security check at Disneyland. In addition, children in the upper grades had higher levels of associative skills. Some children associated the fish in the water, following happily behind them during the night tour, with the little fish in *Finding Nemo*. When viewing the beautiful castle at Disneyland, some children associated it with a princess living an elegant life, just like the ones in the cartoons.

**Table 5:** Statistical analysis of tourist scenes in parent-child tourism.

**Tourist scenes**		**Lower grades**	**Middle grades**	**Upper grades**	**Total**
Realistic tourist scenes	Number of respondents	83	43	55	181
	Percentage	60.58%	55.84%	51.40%	56.39%
Story-based tourist scenes	Number of respondents	41	21	36	98
	Percentage	29.93%	27.27%	33.64%	30.53%
Associative tourist scenes	Number of respondents	14	12	16	42
	Percentage	10.22%	15.58%	14.95%	13.08%

### Experiences with tourism colors

Color is an essential element of the world, and children's perception, discrimination, and choice of things are mostly based on visual perception. Tourism colors refer to children's chromatic impressions of various things in tourist destinations based on their personal experiences and memory information, which are the specific colors that emerge in their drawings. The colors in children's drawings represent not only their preference for particular colors but also the colors the children visually processed at the tourist destination. Data on the colors of the drawings revealed that the colors preferred by children were, in descending order, red (68%), yellow (64.8%), blue (66.4%), orange (56.0%), green (56.0%), purple (34.0%), black (31.20%), and gray (6.80%).

Children are attracted to bright and vibrant warm colors, such as red, yellow, and orange. At the same time, the proportion of cool colors, such as blue and green, is also large because children prefer to use realistic colors when depicting the blue sea, blue sky, green trees, and green jungles. Therefore, the first feature of travel colors depicted by children is brightness. Bright colors are more stimulating to children perceptual nerves. Children are more resistant to grayish colors, such as black and gray.

The second feature of travel colors is authenticity. Children's perception of colors in the tourist contexts is more oriented to the color properties of things themselves, such as the sea being blue, the beach being yellow, and the trees being green. However, it should be noted that such authenticity is limited to the depiction of landscapes. When children depict people on trips or in vehicles while traveling, they paint in bright and warm colors. The color tone and choice of children's drawings provide a cognitive and emotional basis for children to create color images of tourist destinations in their minds.

## Conclusions and implications

### Conclusions

The analysis of 321 children's drawings revealed the main cognitive contents of children's parent-child tourism experiences, including perceptions of tourism elements, perceptions of people, experiences of tourist activities, experiences of tourist scenes, and experiences with tourism colors. Moreover, the parent-child tourism experience can stimulate children's sense of ownership by reversing their roles and allowing them to take charge of their family tours as their parents do, which has a positive effect on the cultivation of children's leadership. In terms of perception of tourism elements, children's perceived elements transform from macroscopic to microscopic, and the perceived content transitions from concrete to abstract (i.e., from common elements, such as people and landscapes to detailed elements, such as modes of travel, food, tourism symbols, etc.). In terms of the perception of persons in travel, children have an acute perception of people, and the actions of the people children meet while traveling can influence their self-perception and tourism experiences. More than 80% of children in our study included people in their drawings, with family members being the most prominent other, followed by friends or partners. In terms of tourist activities, children prefer to draw on the activities in which they participated during their travels.

The types of activities in which children are involved are divided into the categories of recreation and leisure, sightseeing and exploration, and cultural and historical. As children progressed through the grades, their preferred activities shifted from simple recreation and leisure activities to participatory, experiential, knowledge-based activities involving sightseeing and exploration, as well as cultural and historical appreciation. In terms of tourist scenes, children's descriptions of parent-child tourism experiences were dominated by realistic scenes followed by story-based scenes, with a minimum of associative scenes. The realistic scenes were found mainly in the lower grades, while the story-based and associative scenes were found mainly in the upper grades. Elementary school students rely on realistic, story-based, and associative scenes to perceive things. In terms of color experience in tourism, on the one hand, children like warm colors, and bright colors are more stimulating to children's perceptual nerves. Children are more resistant to grayish tones. Moreover, when depicting people, vehicles, symbols, and other elements, children prefer bright and warm colors. On the other hand, children's perception of the colors in the tourist context is more oriented to the realistic colors of the tourist destination itself, such as blue sea, blue sky, and green trees and jungles.

### Theoretical implications

In order to echo the existing research on children's right to speak (49, 50), this study recognizes the special significance of children's parent-child tourism world, which is no longer limited to the way that adults replace children's voices, and uses children's drawings to project children's perceptions of the parent-child tourism experience. At the same time, this study identifies the cognitive rules of children's parent-child tourism experience. The results are helpful in understanding children's tourism psychology under different tourism situations. Compared with the existing studies on children's tourism experiences, such as Wu et al. (15), Kozak (51), and Therkelsen and Lottrup (52), this study places the scenarios of children's tourism experience into the context of family culture, focusing on the psychological processes and emotional cognition of children's tourism experiences in parent-child tourism.

As a beneficial addition to the children's tourism literature in the parent-child setting, this study addresses the fact that mentally immature children cannot convey a complete inner schema to adults. On the one hand, similar to Wang et al. (31), this study supports the idea that the tourism elements perceived by children change from concrete objects to abstract symbols by age. As such, children of different ages have different tourism needs. On the other hand, this study finds that parent-child activities are spiritual center for parent-child tourism experiences, allowing children to gain benefits in the form of skills, social interaction, and self-realization, among other things. Such findings can prompt future researchers to pay attention to the path of the formation of family psychological benefits to children in the context of parent–child tourism and thus enrich children's tourism theory.

### Practical implications

“We see only what we want to see, and we build only what we want to build.” Parents, travel agencies, and government departments have failed to understand the parent-child tourism experience from the children's perspective. Because of this, they only know the parent-child tourism experience superficially, and do not dig deeper into its value or connotation. Thus, the profit-oriented aspect of the market has led to a wide range of parent-child tourism products of varying quality. These products can hardly meet children's demands for experiences.

Based on these factors, we propose practical measures to improve the parent-child tourism experience from the children's perspective. First, appropriate parent-child tourism programs can be designed with the characteristics of children's experiences. Most children in the lower elementary grades have a more centralized focus on tourism and are interested in and vividly remember a particular tourist attraction. For children in this cognitive stage, we should design tourism programs that are participatory and interactive so that children can clearly perceive external information, quickly process it cognitively, and gain a quality tourism experience. Children in the middle and upper grades have already acquired certain abstract thinking skills and have a good perception of abstract symbols, such as travel routes and slogans; they are beginning to think logically. Therefore, for children in the middle and upper grades, the tourist routes should be well-designed, and the tourist scenes can be relatively complex to meet the higher cognitive needs of these children.

Second, children's parent-child tourism experiences can be enhanced by introducing novel activities. The world, in children's eyes, is filled with activities that are novel and enjoyable for children (53), which can bring them a sense of novelty and joy and, in turn, make them surprised and delighted. Compared to adults, child travelers have a stronger curiosity and desire to explore and a different understanding of novelty. Moreover, children construct their own cognitive worlds with a limited stock of information. Novel activities on trips are of great significance to children as they inspire them in terms of imagination, initiative, and leadership.

## Limitations and future studies

This study has several limitations. First, the findings are based on data collections derived from children living in Guangdong, a coastal and developed province at the southern tip of China. Although the study sample includes children in rural areas of Guangdong, due to the uneven development of Eastern and Western China, these findings may not be representative of Chinese children in the northeastern provinces or others. Future research could obtain data from different provinces for comparative research to reach a more complete understanding. We encourage the acquisition of tourism experience data from children of multi-cultural backgrounds and at different times to improve the external validity of our conclusions.

Second, with the introduction and spread of the concept of early education abroad and the transformation of modern family leisure time, the parent-child tourism market is becoming increasingly younger (54, 55), and many parents are giving their children the opportunity to travel and see the world. Future research could consider including children in the research scope, expanding the research object of parent-child tourism experiences to children with less mature cognition, and making the research scope of parent-child tourism experience more extensive. Future research could also extend further to the tourism experiences of preschool children and use technical measurement methods to compare and analyze the tourism experiences of children of different ages.

Finally, the cross-sectional data collected at one time can hardly be used to infer the change process of children's tourism experiences. In the future, longitudinal tracking research could be used to explore the dynamic changes in these experiences.

## Data availability statement

The raw data supporting the conclusions of this article will be made available by the authors, without undue reservation.

## Ethics statement

The studies involving human participants were reviewed and approved by the Secretariat of Academic Committee, Guangdong Polytechnic Normal University. The participants provided their written informed consent to participate in this study.

## Author contributions

XG made substantial contributions and participated in all aspects of the paper, conducted the methodology, analyzed the data, and wrote the manuscript. TL made substantial contributions to the work and participated in all aspects of the paper. All authors read and approved the final manuscript, contributed to the article, and approved the submitted version.

## Funding

This work was supported by the University-level Scientific Research Project of Guangdong Polytechnic Normal University (grant number 2022SDKYB005). The funder had no role in the study design, collection, data analysis, or interpretation of the data, in the writing of the report, or in the decision to submit the article for publication.

## Conflict of interest

The authors declare that the research was conducted in the absence of any commercial or financial relationships that could be construed as a potential conflict of interest.

## Publisher's note

All claims expressed in this article are solely those of the authors and do not necessarily represent those of their affiliated organizations, or those of the publisher, the editors and the reviewers. Any product that may be evaluated in this article, or claim that may be made by its manufacturer, is not guaranteed or endorsed by the publisher.
